# The nanostructuring of surfaces and films using interference lithography and chalcogenide photoresist

**DOI:** 10.1186/s11671-015-0765-y

**Published:** 2015-02-27

**Authors:** Viktor Dan’ko, Ivan Indutnyi, Victor Myn’ko, Mariia Lukaniuk, Petro Shepeliavyi

**Affiliations:** V. Lashkaryov Institute of Semiconductor Physics, National Academy of Sciences of Ukrain, 45, Prospect Nauky, 03028 Kyiv, Ukraine

**Keywords:** 42.70.Ln, 81.16.Rf, Chalcogenide vitreous semiconductors, Photoresist, Photolithography, Photoinduced changes

## Abstract

The reversible and transient photostimulated structural changes in annealed chalcogenide glass (ChG) layers were used to form interference periodic structures on semiconductor surfaces and metal films. It was shown that negative-action etchants based on amines dissolve illuminated parts of a chalcogenide film, i.e., act as positive etchants. The diffraction gratings and 2-D interference structures on germanium ChGs - more environmentally acceptable compounds than traditionally used arsenic chalcogenides - were recorded, and their characteristics were studied.

## Background

Chalcogenide vitreous semiconductors, or, in other words, chalcogenide glasses (ChGs), attract attention of many researchers owing to widely diverse photoinduced changes in their structure and, as a consequence, in their properties (optical characteristics, conductivity, solubility in selective etchants, and even mechanical characteristics). These modifications serve as a basis for the practical use of ChGs as inorganic photoresists, media for optical and electrical information recording, as well as other applications.

Photoinduced modifications in ChG films deposited using thermal evaporation in vacuum have two components: reversible and irreversible [1-3]. In addition, investigations performed *in situ* under exposure to light have shown [4,5] that transient photoinduced structural changes are also observed in ChG films. These changes are only observed during illumination of the films and rapidly relax after switching off the light. It was previously shown that all these modifications (reversible, irreversible, and transient) result in changes of ChG solubility in some solutions and can serve as a base for photoresist process in ChGs [6-9].

Earlier, the use of chalcogenide films as a photoresist was related specifically with irreversible changes in their solubility. ChG films, thermally deposited in vacuum, have a considerable amount of ‘anomalous’ homopolar bonds, also with availability of pores and voids, even if these films have stoichiometric composition. Illumination or annealing causes polymerization of molecular groups that transform into the basic matrix of a chalcogenide glass, with decreasing the number of homopolar bonds and voids and this circumstance specifically changes the physicochemical properties of ChG films and, in particular, their solubility. Sufficiently high etching selectivity is observed in this process only in arsenic-based chalcogenide films, such as As-S, As-Se, and As-S-Se. Photolithography on the as-deposited un-annealed layers of GhG was described in more detail in [10].

In this paper, we present the results of investigations of photolithography on annealed GhG layers, i.e., photolithography that is based on the reversible and transient photochanges in GhGs.

Chalcogenide photoresists based on thermal evaporated amorphous films of chalcogenide glasses are characterized by high resolution, optical uniformity, wide spectral range of photosensitivity, and the possibility to be used on both planar and non-planar substrates. Moreover, the annealing of ChG films near the glass transition (*T*_g_) temperature results in decreasing surface roughness of these films and allows to use in lithographic process on more environmentally friendly ChGs based on germanium and films deposited using a non-thermal method (laser or magnetron sputtering in vacuum, deposition from solutions) [6,9,11].

In addition, these photoresists possess a high refractive index ranging from 2.0 to 3.0 and are very perspective for immersion lithography [10], including high-resolution interference (interferometric) lithography that is one of the most technological methods for fabrication of periodic nano- and microstructures, production of the master mold for nanoimprinting lithography, and formation of grating structures on semiconductor and other surfaces.

## Methods

The samples for our study were prepared using successive thermal vacuum deposition of Cr adhesive layer and ChG layers onto polished glass and silicon substrates at a residual pressure of 2 × 10^−3^ Pa. For the nanostructuring of some metal films, these layers were deposited directly on the substrate before the Cr and ChG ones. The deposition rate and films thicknesses were monitored *in situ* with a KIT-1 quartz thickness meter. After deposition, the film thicknesses were controlled using a MII-4 microinterferometer. The deposited films were annealed for 0.5 to 2 h at temperatures ranging from *T*_g_ - 15°C to *T*_g_ - 5°C, where *T*_g_ is the glass transition temperature of a given chalcogenide.

The etching rates of ChG films were studied using the quartz oscillator method [9,12] in a quartz cuvette filled with a selective etchant [13] based on an anhydrous solution of amines.

Recording the interference structures on annealed ChG films was carried out using the interference pattern formed with a helium-cadmium laser (wavelength *λ* = 440 nm) of the holographic setup assembled by the wave-amplitude division method. For decreasing the interference pattern period, glass prisms with a refractive index ranging from 1.5 to 2.0 were used. In the case of photoetching, the samples were placed into the quartz cuvette filled with selective etchants during exposure.

The profile shape of the obtained structures and surface roughness of ChG films were investigated with a Dimension 3000 Scanning Probe atomic force microscope (AFM) (Digital Instruments Inc., Tonawanda, NY, USA).

## Results and discussion

**Figure 1:**
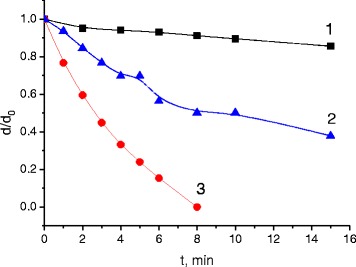
**Dissolution kinetics of Ge**_**25**_ **Se**_**75**_ **layers in the amine-based etchant.** Curve 1 - annealed and unexposed layer, curve 2 - annealed layer exposed before etching by integral radiation of the mercury lamp (250 W) for 45 min, and curve 3 - annealed layer exposed using the same lamp during etching.

Figure 1 shows the kinetic curves *d*(*t*) for the etching of Ge_25_Se_75_ layers in a selective amine-based etchant [13]. Ge_25_Se_75_ composition, which lies within the range of the intermediate phase glass compositions [14], was chosen as an object of our study. The initial thickness of these layers was *d*_0_ = 300 nm (*d* is the residual layer thickness after etching, and *t* is the duration of etching).

It can be seen in Figure 1 that the selected non-water etchant based on amine solution is characterized by good selectivity for the annealed Ge_25_Se_75_ layer (the value of selectivity defined by the ratio of the dissolution rates for exposed and unexposed layers reached 20). But in contrast to traditional lithography on ChG photoresists that use thermally deposited (and non-annealed) ChG layers, and where negative selectivity takes place (unexposed photoresist areas are dissolved faster than the exposed ones), in our case, the layers that illuminated in the process of etching (curve 3, as compared with curve 1) or those that illuminated before etching (curve 2, as compared with curve 1) dissolve faster.

Similar results with certain quantitative details were obtained for arsenic chalcogenide films, but for the arsenic-based ChG films, the photoetching effect occurs only on annealed layers, while on germanium-based ChGs, this effect is pronounced in un-annealed layers, too. The possible mechanism of the photoinduced etching effect was discussed in our previous paper [11].

**Figure 2:**
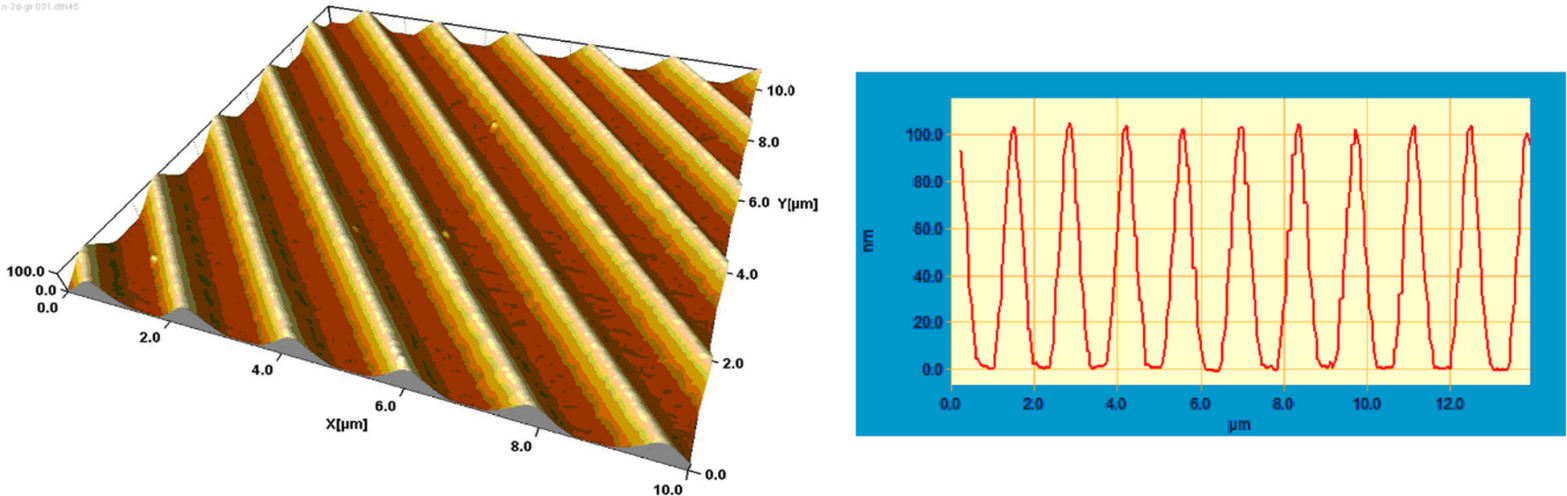
**AFM image and profile of a diffraction grating.** AFM image and profile of a diffraction grating recorded in Ge_25_Se_75_ layer by using the method of interference lithography with photoinduced etching and the profile of its grooves.

Figure 2 shows the AFM image of a diffraction grating recorded in Ge_25_Se_75_ layer by using the method of interference lithography with photoinduced etching as well as the profile of its grooves. The spatial frequency of grooves in the recorded grating is 740 mm^−1^, and the profile depth is 200 nm. The shape of the groove profile of the grating is not sinusoidal, which points out that the time of photoetching process was greater than the optimal one.

**Figure 3:**
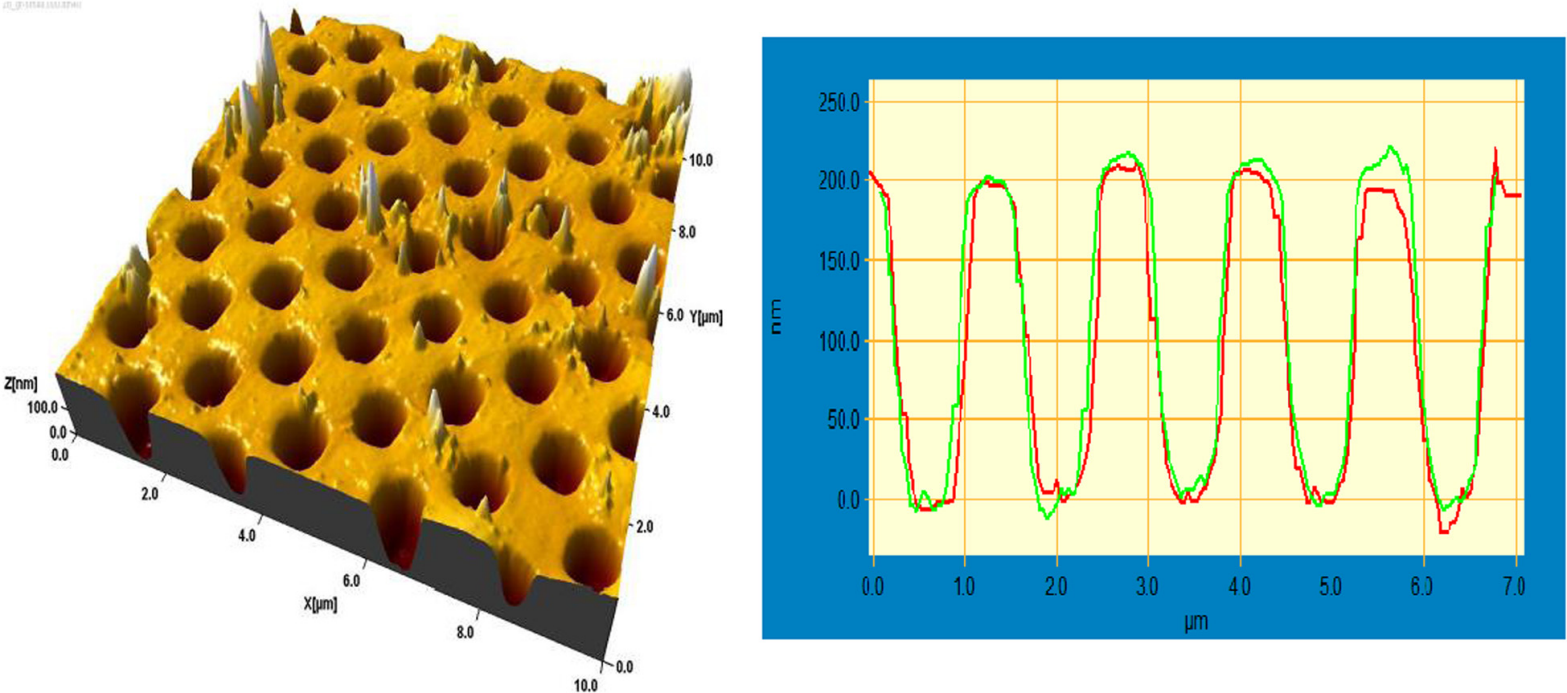
**AFM image of macro-porous silicon obtained by metal-assisted etching and diagonal section profile of the sample.**

The interference lithography method allows forming solid surface structures of more complicated configurations than gratings by multiple exposures to interference patterns of more than two coherent beams or by exposure to a complex interference pattern formed by multiple coherent beams. We have combined this method with the method of the metal-assisted chemical etching [15,16] of silicon to obtain macro-porous samples. With this aim, Au layer (20 nm) and chalcogenide (As_40_S_30_Se_30_) layer (100 nm) were deposited onto the Si plate. By using interference lithography and the photoetching method, a chalcogenide mask was created, which enabled us to etch the Au layer. Then, remains of chalcogenide were removed, and the silicon plate was etched in the solution HF/H_2_O_2_. The areas of silicon that had a contact with metal in this chemical etching process were dissolved. The final picture is shown in Figure 3. The depth of the obtained cavities is rather great, and their profile is near rectangular.

In the annealed ChG films, a small reversible photostimulated structural changes are observed, which can be reversed by annealing near the glass transition temperature. In our previous investigation, it was shown that the reversible structural changes are also accompanied by a change in the solubility of ChG films, and amine-based solution acts as a positive etchant [6,17]. Noticeable selectivity occurs in annealed film based on germanium chalcogenide, too (see, for example, curves 1 and 2 in Figure 1), which is more environmentally friendly than arsenic compounds. By optimization of etchant composition and value of exposure, this selectivity can be enhanced (up to 10), and interference lithography on germanium chalcogenide can be realized.

Below, we present the results of comparative investigations of the reversible changes and positive resistive process on annealed germanium-based ChG films.

Interference structures on the annealed Ge_25_Se_75_ films (parts 1 and 2) were recorded by their exposure to an interference pattern. After exposure, one part (1) of the exposed sample was repeatedly annealed under the same conditions then both parts (1 and 2) were etched in amine-based etchant. As a result, the diffraction structure with given parameters was obtained on the part 2, but no pattern were formed on the part 1. These exposure-annealing cycles can be repeated several times without changes in their parameters.

**Figure 4:**
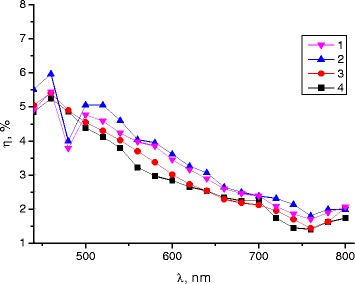
**Diffraction efficiency of interference structures.** Spectral dependence of the diffraction efficiency *η* (in unpolarized light) for the gratings recorded on annealed Ge_25_Se_75_ films: curves 1, 2, 3, and 4 correspond to samples recorded after the first, second, third, and fourth exposure-annealing cycles, accordingly.

Figure 4 shows the diffraction efficiency of these interference structures recorded on annealed Ge_25_Se_75_ layers after exposure-annealing cycles that were repeated under the same conditions several times. As seen, the diffraction characteristics and surface morphology of the recorded diffraction structure do not change from cycle to cycle.

The results illustrate that the investigated resistive process on annealed Ge_25_Se_75_ films is specifically related with reversible photostructural changes that can be reversed by annealing.

The result of this experiment allowed us to develop the new photolithographic method on annealed ChG films. The difference of this method from photoetching consists in the sequence of exposition and etching processes.

**Figure 5:**
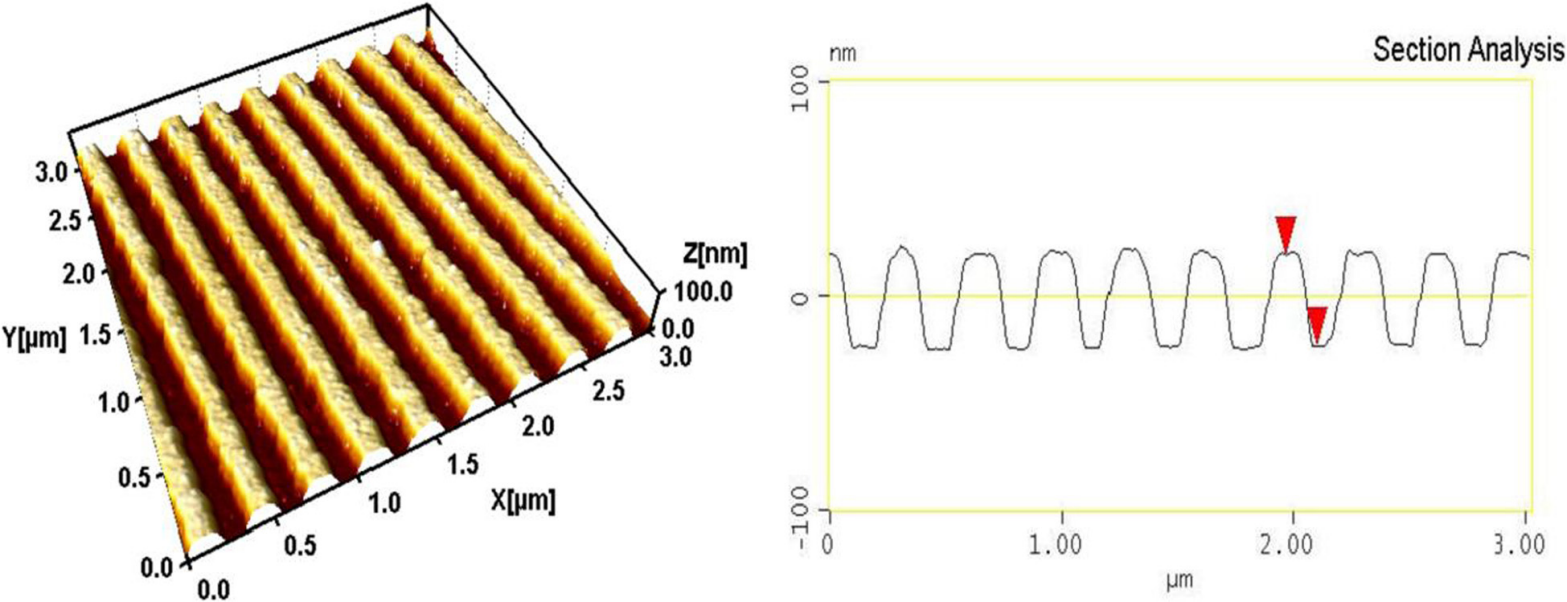
**AFM image and profile of the Cr nanowires on the surface of a silicon wafer.**

**Figure 6:**
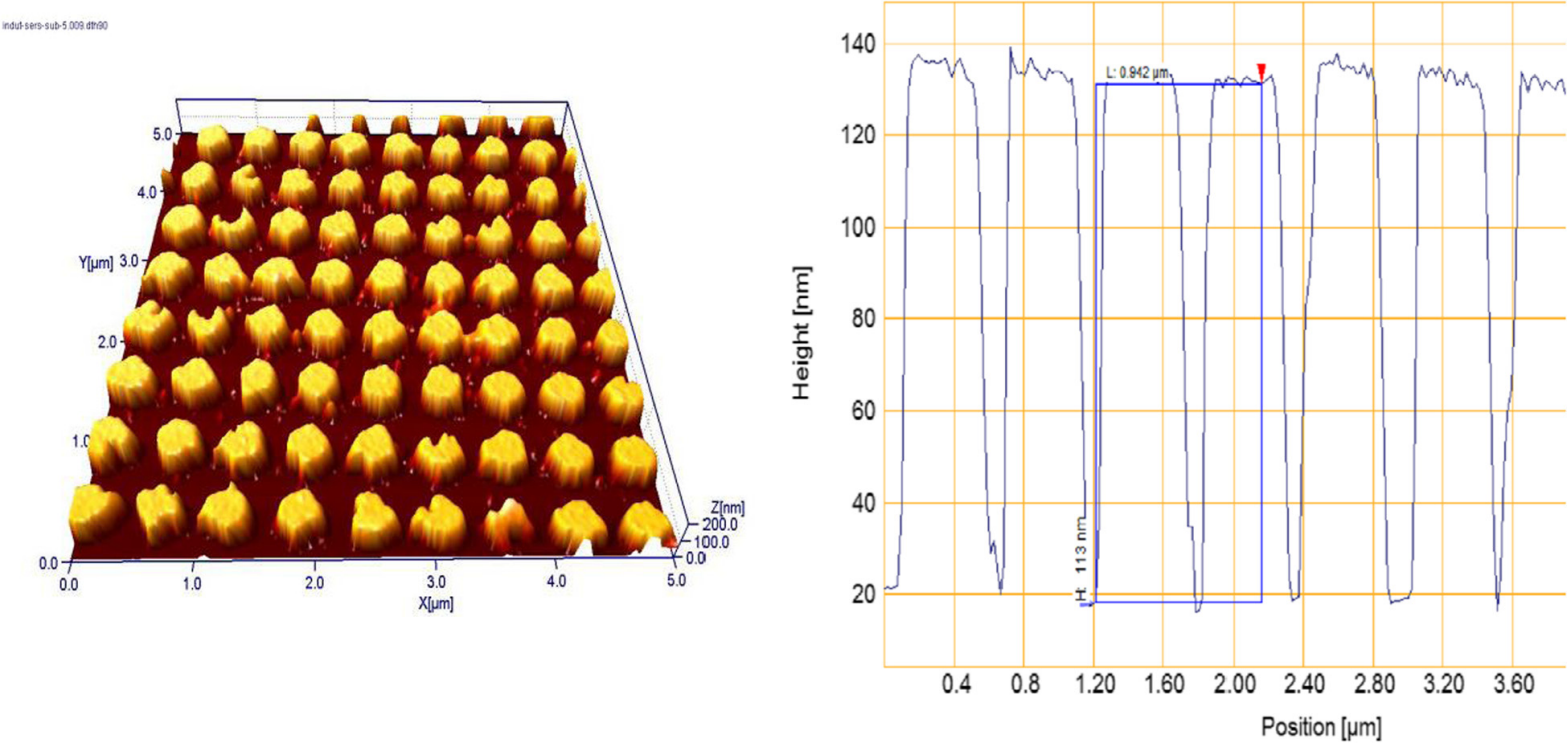
**AFM image and profile of the 2-D Au structure on a glass plate.**

This technology has been used for fabrication of one- and two-dimensional periodic structures with a spatial frequency up to 5,000 mm^−1^. As an example, Figures 5 and 6 present the AFM image of the Cr nanowires (period - 330 nm, width of the wire - about 120 nm, thickness - 40 nm) on the surface of silicon wafer and 2-D Au structure on a glass plate. Both samples are produced using interference lithography on annealed Ge_25_Se_75_ films and etching of the metal layers through a chalcogenide mask. The obtained high-frequency structures are used in optochemical sensors based on plasmonic systems.

## Conclusions

The first studies of the interference photolithography on annealed GhG layers, which is based on the reversible and transient photostimulated structural changes in GhG films, were performed.

The developed methods have several advantages in comparison with the existing methods of interference lithography on chalcogenide photoresists.

Such lithography can be performed on annealed chalcogenide layers, which reduces the surface roughness of products after selective etching. This makes it possible to obtain lithographic masks and periodic relief-phase structures of higher quality.

In this technology, we have used germanium ChGs - non-toxic, more environmentally acceptable compounds than traditionally used arsenic chalcogenides.

The studied technique used to form interference periodic structures onto annealed germanium chalcogenide layers is simple, inexpensive, and adaptable to large-scale manufacturing.

## Abbreviations

AFM: atomic force microscope
ChGs: chalcogenide glasses
